# A Pilot Study to evaluate feasibility of a teleneuropsychological protocol in older adults in Panama and Florida

**DOI:** 10.1002/alz.090965

**Published:** 2025-01-03

**Authors:** Luis Guillermo Santos, Ambar R Perez‐Lao, Diana C Oviedo, Gabrielle B Britton, Glenn E. Smith

**Affiliations:** ^1^ Instituto de Investigaciones Científicas y Servicios de Alta Tecnología (INDICASAT AIP), Panama Panama; ^2^ Universidad Santa Maria La Antigua, Panama Panama; ^3^ University of Florida, Florida, FL USA; ^4^ Instituto de Investigaciones Científicas y Servicios de Alta Tecnología (INDICASAT AIP), Centro de Neurociencias, Panamá, Panamá Panama; ^5^ Sistema Nacional de Investigación (SNI), Panamá, Panamá Panama; ^6^ Universidad Santa María La Antigua, Panamá, Panamá Panama; ^7^ 1Florida Alzheimer’s Disease Research Center, Gainesville, FL USA; ^8^ Mayo Clinic, Rochester, MN USA; ^9^ University of Florida, Gainesville, FL USA

## Abstract

**Background:**

The use of videoconference platforms for neuropsychological assessment was not as common among mental health practitioners before the COVID‐19 pandemic. However, due to lockdowns and quarantines worldwide, mental health professionals had to find a feasible alternative and shift to virtual evaluations. This increased the use of teleneuropsychology in both at a clinical and research level. Studies conducted during the pandemic provided evidence of the feasibility of virtual assessments, but data from low‐and middle‐income countries is scarce and conclusions from studies in other regions cannot be generalized. The objective of this study is to develop and apply a teleneuropsychological assessment battery on a group of elderly adults in Panama and Hispanics living in the United States.

**Method:**

This is a multicenter study that will gather data from a Panamanian Cohort parallel to the current Florida‐based study Development and Validation of TeleNeuropsychology Normative Data for Older Adults (FLOAT). So far 80 participants over 50 years old have been assessed in Panama and Florida. Inclusion criteria includes passing a screening with TICS‐M for low risk of developing dementia and having basic knowledge and accessibility of electronic devices. Researchers contact potential participants through calls and WhatsApp chats to explain the study and obtain informed consent. Participants are then screened to see if they are eligible to be assessed through the video conference platform. Participants must complete the teleneuropsychological assessment battery from the National Alzheimer’s Coordinating Center (NACC), and different questionnaires to provide socio‐demographic and functional data. All information is gathered with the REDCap platform.

**Result:**

One hundred participants will be assessed with a teleneuropsychology protocol in each cohort. Once all data is collected, it will be analyzed with multivariate statistics methods. We expect to describe the protocol’s feasibility assessing older adults in both locations.

**Conclusion:**

To our knowledge this is the first study of its kind in Panama and one of the only ones in the region. The study seeks to generate data that could be shared and applied for future research or clinical practice regarding teleneuropsychological assessments with older adults with similar cohorts.

